# A Synthetic Tetramer of Galectin-1 and Galectin-3 Amplifies Pro-apoptotic Signaling by Integrating the Activity of Both Galectins

**DOI:** 10.3389/fchem.2019.00898

**Published:** 2020-01-10

**Authors:** Shaheen A. Farhadi, Margaret M. Fettis, Renjie Liu, Gregory A. Hudalla

**Affiliations:** J. Crayton Pruitt Family Department of Biomedical Engineering, University of Florida, Gainesville, FL, United States

**Keywords:** galectin-1, galectin-3, glycobiology, protein engineering, protein-carbohydrate binding

## Abstract

Galectin-1 (G1) and galectin-3 (G3) are carbohydrate-binding proteins that can signal apoptosis in T cells. We recently reported that a synthetic tetramer with two G1 and two G3 domains (“G1/G3 Zipper”) induces Jurkat T cell death more potently than G1. The pro-apoptotic signaling pathway of G1/G3 Zipper was not elucidated, but we hypothesized based on prior work that the G1 domains acted as the signaling units, while the G3 domains served as anchors that increase glycan-binding affinity. To test this, here we studied the involvement of different cell membrane glycoproteins and intracellular mediators in pro-apoptotic signaling via G1/G3 Zipper, G1, and G3. G1/G3 Zipper induced Jurkat T cell death more potently than G1 and G3 alone or in combination. G1/G3 Zipper, G1, and G3 increased caspase-8 activity, yet only G1 and G3 depended on it to induce cell death. G3 increased caspase-3 activity more than G1/G3 Zipper and G1, while all three galectin variants required it to induce cell death. JNK activation had similar roles downstream of G1/G3 Zipper, G1, and G3, whereas ERK had differing roles. CD45 was essential for G1 activity, and was involved in signaling via G1/G3 Zipper and G3. CD7 inhibited G1/G3 Zipper activity at low galectin concentrations but not at high galectin concentrations. In contrast, CD7 was necessary for G1 and G3 signaling at low galectin concentration but antagonistic at high galectin concentrations. Collectively, these observations suggest that G1/G3 Zipper amplifies pro-apoptotic signaling through the integrated activity of both the G1 and G3 domains.

## Introduction

Galectins are a family of soluble carbohydrate-binding proteins that regulate cell phenotype and function in development and disease (Cummings et al., 2015). For example, galectins are integral to fetal-maternal tolerance, mediators of cell-cell and cell-matrix adhesion, prevent the onset and progression of various autoimmune diseases, activate pro-inflammatory responses during osteoarthritis, can enhance or inhibit pathogen entry into host cells, and confer immune privilege to various tissues as well as tumors (Rabinovich et al., 1999; Hughes, 2001; Santucci et al., 2003; St-Pierre et al., 2011; Than et al., 2012; Li et al., 2013; Baum et al., 2014; Hu et al., 2017). Galectin-1 (G1) and galectin-3 (G3) are expressed by many immune cells and receive considerable attention in the context of immunity (Rabinovich and Toscano, 2009; Thiemann and Baum, 2016). They can act on various cell types, including monocytes, macrophages, neutrophils, dendritic cells, and T cells to regulate cell adhesion, migration, proliferation, cytokine secretion, or death (Elola et al., 2005; Vasta et al., 2012; Chung et al., 2013). Among immune cells, the activity of G1 and G3 on T cells has been studied most extensively. Notably, G1 and G3 demonstrate similar biological activities toward T cells in some contexts, yet divergent activity in others. For example, both G1 and G3 can induce T cell apoptosis (Stillman et al., 2006), whereas only G3 has been shown to induce T cell secretion of interleukin-2 (Hsu et al., 1996). Further, G1 can stimulate antigen-specific T cell responses, whereas G3 failed to stimulate these responses (Tribulatti et al., 2012). Moreover, G3 induced activated human T cell death, whereas G1 did not, instead promoting immunosuppressive interleukin-10 production and suppressing interferon-γ secretion (Stowell et al., 2008b). Thus, understanding the pathways by which G1 and G3 evoke changes in immune cell behavior, as well as any interplay between them, presents opportunities to regulate innate and adaptive immune responses.

Both G1 and G3 activate apoptosis of T cells by crosslinking glycoproteins displayed on the plasma membrane, yet evidence suggests that they may signal through distinct intracellular pathways. For example, early reports suggested that G1 induces apoptosis in a caspase- and cytochrome c-independent manner through nuclear translocation of endonuclease G (Hahn et al., 2004). More recently, it has been shown that G1 can induce Jurkat T cell death due to activation of c-Jun N-terminal kinase (JNK) which, in turn, activates c-Jun and AP-1 (Brandt et al., 2010). G1 has also been reported to sensitize resting human T cells to Fas-mediated cell death (Matarrese et al., 2005), as well as induce Fas-dependent apoptosis of Jurkat T cells via activation of caspase-8 and caspase-3 (Brandt et al., 2008). The pathways through which extracellular G3 induces apoptosis are less understood at present. Early reports demonstrated that extracellular G3 signals apoptosis via cytochrome c-release and caspase-3 activation independent of caspase-8 activation (Fukumori et al., 2003), with more recent data suggesting that G3 activates caspase-9 upstream of caspase-3 through phosphorylation of extracellular signal-regulated kinase (ERK) (Xue et al., 2017).

Differences in the pro-apoptotic signaling pathways activated by extracellular G1 and G3 may arise because they recognize different cell surface glycoproteins by way of their selectivity for different oligosaccharide ligands (Stowell et al., 2008a). Among glycoprotein receptors implicated in T cell death (e.g., CD7, CD29, CD43, CD45, and CD71) (Brown et al., 1996; Lesage et al., 1997; Lesnikov et al., 2001; Arencibia et al., 2002), apoptosis induced by G1 has been suggested to depend on CD7 but not CD45 (Pace et al., 1999, 2000; Walzel et al., 1999; Stillman et al., 2006), whereas other studies have identified a role for CD45 in G1 pro-apoptotic signaling (Nguyen et al., 2001). In contrast, apoptosis via extracellular G3 has been suggested to depend on CD45 but not CD43, whereas conflicting roles of CD7 and CD29 have been reported (Fukumori et al., 2003; Stillman et al., 2006). Further, CD71 was observed to cluster on dying cells treated with G3, whereas G1 cannot bind CD71 (Stillman et al., 2006).

**Figure 1:**
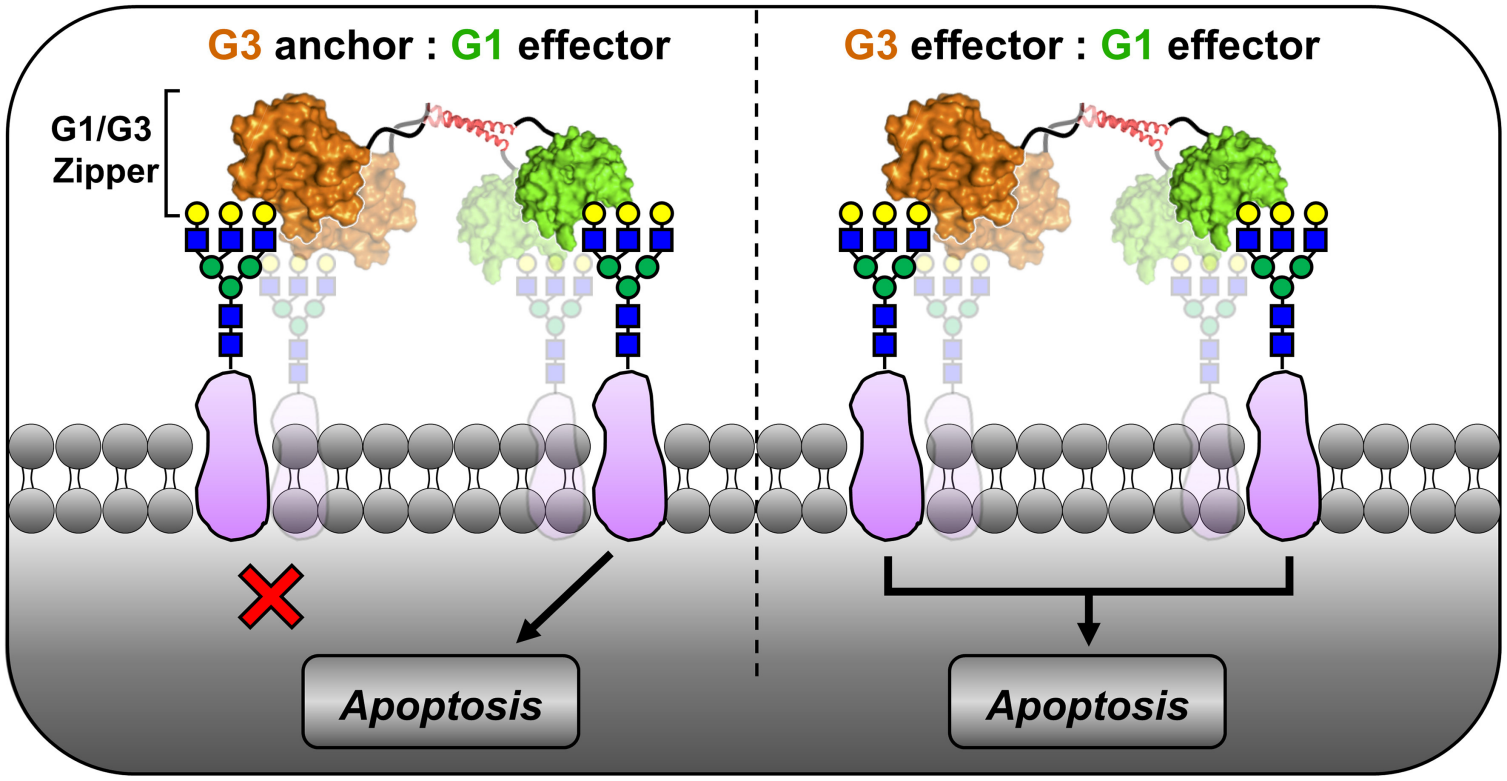
Proposed models of G1/G3 Zipper pro-apoptotic signaling activity. In one model, G3 binding to membrane glycoproteins anchors G1 at the cell surface, where G1 acts as the only signaling domain **(Left)**. In an alternative model, both G1 and G3 act as signaling domains to amplify apoptosis **(Right)**.

Combination therapies that engage different receptors or pathways can be used to regulate T cell responses by amplifying apoptosis and/or modulating activation (Strauss et al., 2002; Reddy et al., 2012). Intriguingly, the combination of native G1 and G3 was neither additive nor synergistic for activating T cell apoptosis despite engaging different receptors and distinct pathways (Stillman et al., 2006). Consistent with this, we previously reported that a fusion protein consisting of G1 linked to the N-terminus of G3 (i.e., “G1/G3”) failed to induce Jurkat T cell apoptosis; however, a synthetic tetramer developed by engineering G1/G3 to self-assemble (i.e., “G1/G3 Zipper”) induced apoptosis at a significantly lower concentration than G1 alone (Fettis et al., 2019). We hypothesized that the G1 domains acted as the signaling units, while the G3 domains served as anchors that increase the apparent glycan-binding affinity (Figure 1, left). This was based on two previous observations. First, G1 fixed in a homodimeric configuration has increased pro-apoptotic signaling activity when compared to native G1, regardless of whether the dimeric structure is established by a peptide linker, α-helical coiled-coil, Fc fusion, or synthetic polymer (van der Leij et al., 2007; Cedeno-Laurent et al., 2010; Earl et al., 2011; Farhadi and Hudalla, 2016; Fettis and Hudalla, 2018). Second, a synthetic homotrimer of G3 lacked signaling activity when compared to native G3 (Farhadi et al., 2018), likely because its carbohydrate-recognition domain valency was insufficient to activate intracellular signaling pathways to a measurable extent.

Here, we sought to determine whether the G1 domains of G1/G3 Zipper act as signaling units while G3 domains serve as anchors (Figure 1, left), or if both galectin domains of G1/G3 Zipper act as signaling units (Figure 1, right). To this end, we studied the involvement of different cell membrane receptors and intracellular mediators in pro-apoptotic signaling via G1/G3 Zipper, G1, and G3. In particular, we evaluated the role of caspase activity (e.g., caspase-8 and caspase-3), as well as ERK and JNK activation. Additionally, we used CD7- and CD45-deficient T cell lines to assess G1/G3 Zipper-, G1-, and G3-induced transmembrane signal transduction.

## Materials and Methods

### Protein Expression and Purification

All proteins in this study were expressed from recombinant DNA in Origami™ B(DE3) Competent *E. coli* (70837-4, Novagen) and purified according to established protocols (Fettis et al., 2019). Protein sequences of G1, which has been mutated to lack surface cysteines, G3, and G1/G3 Zipper have been published elsewhere (Restuccia et al., 2018; Fettis et al., 2019). After purification, molecular weight and purity of each protein were determined via denaturing gel electrophoresis and Coomassie staining. Molar concentration of each purified protein was determined using the Pierce™ 660 nm Protein Assay Reagent (22660, ThermoFisher). Finally, endotoxin content was reduced to <1 EU/mL via Triton X-114 cloud-point precipitation and then confirmed using the Pierce™ Chromogenic Endotoxin Quantitation kit (A39552, ThermoFisher), according to manufacturer instructions.

### Cell Death Assays

Protocols for flow cytometric analysis of apoptosis were adapted from previously reported methods (Pace et al., 2003). Jurkat E6-1 (ATCC^®^ TIB-152™), HuT 78 (ATCC^®^ TIB-161™), and J45.01 (ATCC^®^ CRL-1990™) T cells were expanded in complete media (RPMI 1640 supplemented with 10% heat-inactivated fetal bovine serum, 1% penicillin–streptomycin, 200 mM L-glutamine, 1% HEPES buffer) at 37°C, 5% CO_2_. For all apoptosis experiments, 100 μL of cells were aliquoted at 200,000 cells into round-bottom 12 × 75 mm culture test tubes (14-956-3D, ThermoFisher) and incubated with 100 μL of sterile 1x PBS (Hyclone™ SH30256) alone (i.e., untreated), G1, G3, G1 + G3, or G1/G3 Zipper in sterile 1x PBS (final galectin concentration depending on assay) in the presence or absence of 100 μM caspase-8 inhibitor Z-IETD-FMK (FMK007, R&D Systems), caspase-3/7 inhibitor I (218826, MilliporeSigma), ERK inhibitor U0126 (662005, MilliporeSigma), or JNK inhibitor II SP600125 (420119, MilliporeSigma) for 4 or 24 h at 37°C, 5% CO_2_. Note, inhibitors were dissolved in American Chemical Society grade dimethyl sulfoxide (DMSO) and an equivalent amount of DMSO (1 μL or 0.5% final concentration) was added to all groups not receiving inhibitors as vehicle control. Further, cells received inhibitor alone as control to calculate a final percentage of cell death after data were collected. Positive single stain controls for flow cytometric analysis were produced by treating cells with 1 μM (S)-(+)-Camptothecin (C9911, MilliporeSigma) for 4 or 24 h at 37°C, 5% CO_2_. After incubation, half the volume of cells treated with (S)-(+)-Camptothecin was heated to 56°C for 5 min and then cooled on ice for 5 min before being recombined with the other half of (S)-(+)-Camptothecin-treated cells. All cells were treated with 1 mL of ice-cold 100 mM lactose in sterile 1x PBS, then pelleted via centrifugation (500 × *g* for 5 min at 4°C) and resuspended in 1 mL of ice-cold sterile 1x PBS. Cells were then stained with 1 μL (1:1,000 dye:PBS volume ratio) of LIVE/DEAD^®^ Near-IR dye (excitation λ = 633 nm and emission λ = 750 nm) on ice for 30 min while protected from light, according to protocols from a LIVE/DEAD^®^ Fixable Near-IR Dead Cell Stain Kit (L34975, ThermoFisher). After staining, cells were washed with 1 mL of ice-cold 1x PBS via centrifugation and the supernatant was carefully discarded. Cells were then resuspended in 100 μL of 1x Annexin V Binding Buffer (556454, BD Biosciences) with 5 μL BV421 Annexin V (563973, BD Biosciences) to stain for phosphatidylserine exposure, and then mixed gently followed by 15 min incubation at room temperature in the dark, according to manufacturer protocols. Finally, 200 μL of 1x Annexin V Binding Buffer was further added to the cells before flow cytometric data was acquired on a BD FACSCelesta™ flow cytometer equipped with BD FACSDiva™ software, a violet laser (405 nm) for BV421 detection (excitation λ = 407 nm and emission λ = 421 nm), and a red laser (640 nm) for LIVE/DEAD^®^ detection (excitation λ = 650 nm and emission λ = 785 nm). Data were analyzed and graphed as scatter plots using BD FlowJo™ software (version 10.0.7). Percentage of cell death was then calculated as follows: [(% annexin V^−^ and LIVE/DEAD^®−^ cells in untreated group)—(% annexin V^−^ and LIVE/DEAD^®−^ cells in treated group)]/(% annexin V^−^ and LIVE/DEAD^®−^ cells in untreated group). Values less than zero were reported as zero. Percentages of cell death that were positive after treatment with inhibitor alone (Supplementary Figure 2) were, respectively, subtracted from protein plus inhibitor treated groups to yield a final percentage of galectin-induced cell death in experimental groups.

### Caspase Activity

Jurkat T cells were expanded in complete media as described above. Jurkat T cells were aliquoted (500,000 cells/well) into sterile, clear, tissue culture treated 6-well microplates. Cells were then incubated with 5 μM G1, 5 μM G3, or 0.5 μM G1/G3 Zipper for 24 h at 37 °C, 5% CO_2_. Cells were washed with sterile 1x PBS prior to measuring caspase activity with the following commercially available assay kits: FLICE/Caspase-8 Colorimetric Assay Kit (K113100, ThermoFisher) and EnzCheck^®^ Caspase-3 Assay Kit #1 (E13183, ThermoFisher). Note, the substrate Z-DEVD-AMC in the EnzCheck^®^ Caspase-3 Assay Kit #1 can also be activated by caspase-7. Herein, we refer to any measured activity as “caspase-3 activity” in accordance with the manufacturer's naming convention. For caspase-8 activity measurements, groups of cells were pooled together at 1.5 ^*^ 10^6^ cells per replicate.

### Statistical Analysis

All experimental and control groups had *N* = 3, and the data were reported as mean ± standard deviation. Data were analyzed for statistically significant differences using one-way ANOVA with Tukey's *post hoc* (*p* < 0.05) in GraphPad Prism 8.0 (GraphPad Software, San Diego, CA, USA).

## Results

### G1/G3 Zipper Induces Jurkat T Cell Death More Potently Than G1 and G3 Alone or in Combination

**Figure 2:**
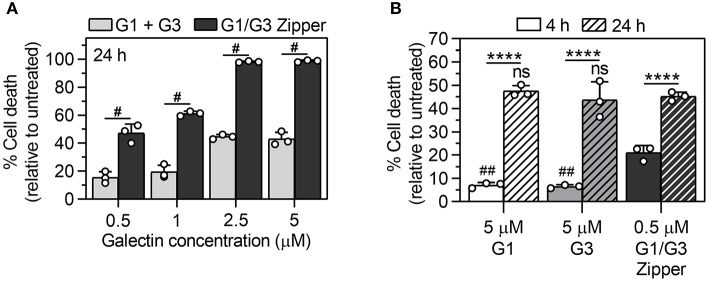
Quantification of Jurkat T cell death via G1/G3 Zipper, G1, G3, or G1 + G3. **(A)** Percentage of dead Jurkat T cells treated with 0.5–5 μM of G1 + G3 (equimolar ratio) or G1/G3 Zipper for 24 h relative to untreated cells. **(B)** Percentage of dead Jurkat T cells treated with 5 μM G1, 5 μM G3, or 0.5 μM G1/G3 Zipper for 4 or 24 h relative to untreated cells. For **(A)**, ^#^indicates significant differences between G1 + G3 and G1/G3 Zipper at each concentration tested and denotes *p* < 0.0001. For **(B)**, *indicates significant differences between time; ^#^indicates significant differences relative to G1/G3 Zipper at *t* = 4 h; “ns” indicates no significant difference relative to G1/G3 Zipper at *t* = 24 h; two symbols denote *p* < 0.01 and four symbols denote *p* < 0.0001. All data shown are *N* = 3, mean ± standard deviation, and tested for statistically significant differences using one-way ANOVA with Tukey's *post-hoc*.

Jurkat T cell apoptosis induced by G1 and G3 is associated with early exposure of phosphatidylserine on the outer leaflet of the plasma membrane, followed by late membrane permeability (Pace et al., 1999, 2003; Stowell et al., 2008b). Here, we used a combination of annexin V staining of phosphatidylserine and a membrane-impermeable DNA-binding dye (LIVE/DEAD^®^, Invitrogen) to quantify Jurkat T cell death induced by G1/G3 Zipper, G1, or G3 as a function of galectin concentration and time. Note, cells were treated with as low as 0.5 μM G1/G3 Zipper in all experiments based on a prior report showing that high concentration of this protein led to loss of cell integrity (Fettis et al., 2019), whereas cells were treated with as high as 5 μM G1 or G3 based on the reported effective dose of these proteins (Restuccia et al., 2015, 2018; Farhadi et al., 2018; Fettis and Hudalla, 2018; Fettis et al., 2019). For each concentration of galectin (0.5, 1, 2.5, and 5 μM) tested, G1/G3 Zipper induced significantly more cell death by 24 h than an equimolar combination of G1 and G3 (Figure 2A). At 4 h, 0.5 μM G1/G3 Zipper induced significantly more cell death than either 5 μM G1 or 5 μM G3 alone (Figure 2B). By 24 h, 5 μM G1 and 5 μM G3 induced a comparable extent of Jurkat T cell death as 0.5 μM G1/G3 Zipper (Figure 2B).

### G1/G3 Zipper, G1, and G3 Differentially Activate Caspase-8 and Caspase-3

**Figure 3:**
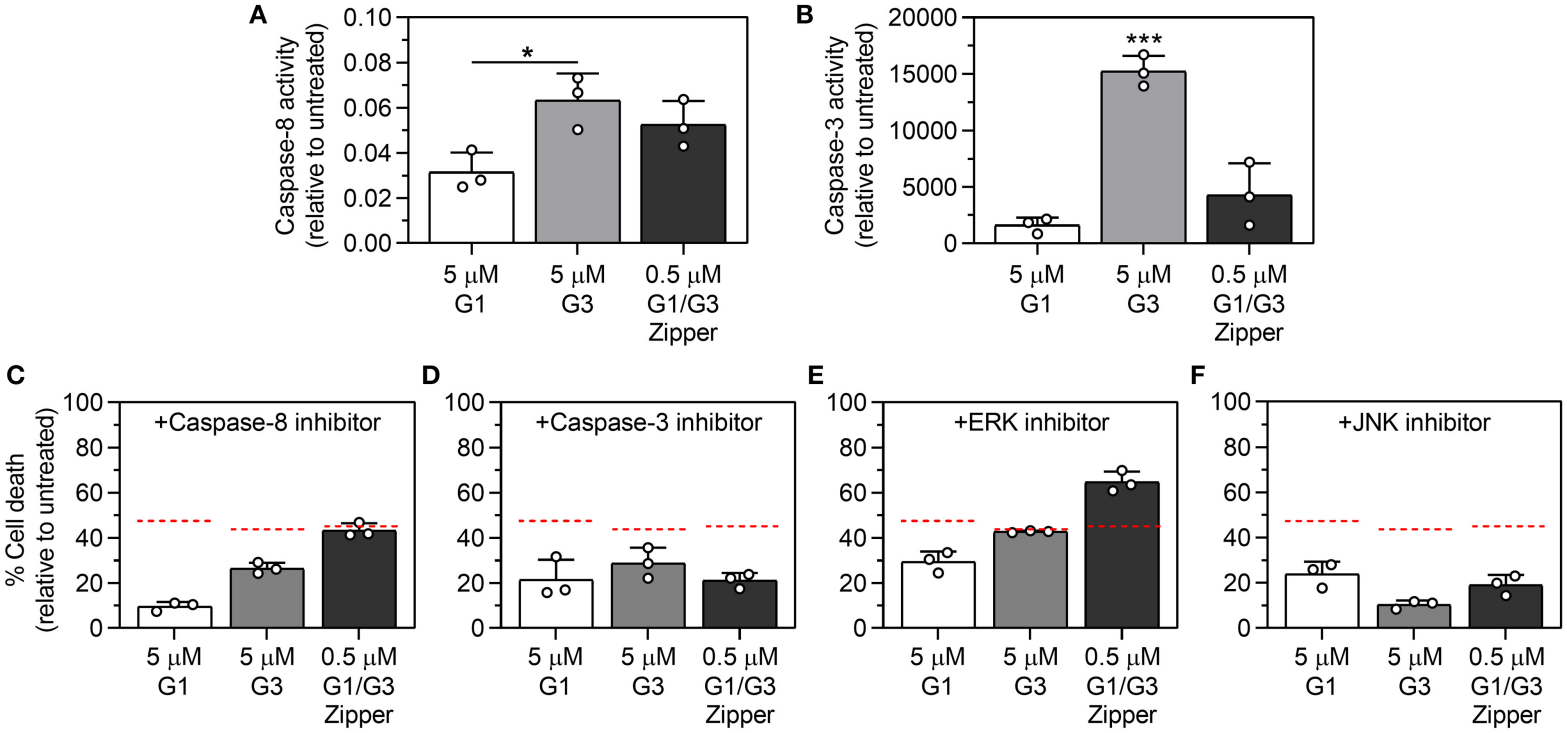
Characterization of the role of caspase-8, caspase-3, ERK, and JNK activation in Jurkat T cell apoptosis induced by G1/G3 Zipper, G1, or G3. **(A)** Quantification of caspase-8 activity and **(B)** quantification of caspase-3 activity in Jurkat T cells treated with 5 μM G1, 5 μM G3, or 0.5 μM G1/G3 Zipper for 24 h relative to untreated cells. Percentage of dead Jurkat T cells (relative to untreated cells) following treatment with 5 μM G1, 5 μM G3, or 0.5 μM G1/G3 Zipper for 24 h in the presence of **(C)** caspase-8 inhibitor (Z-IETD-FMK), **(D)** caspase-3 inhibitor I, **(E)** ERK inhibitor (U0126), or **(F)** JNK inhibitor II (SP600125). For comparison, the red dashed lines in **(C–F)** indicate the percentage of dead Jurkat T cells following treatment with 5 μM G1, 5 μM G3, or 0.5 μM G1/G3 Zipper for 24 h, respectively, from Figure 2B. For **(A,B)**, * indicates significant differences between groups; one symbol denotes *p* < 0.05, three symbols denote *p* < 0.001; statistically significant differences were determined using one-way ANOVA with Tukey's *post-hoc*. All data shown are *N* = 3, mean ± standard deviation.

We characterized caspase-8 activity induced by G1/G3 Zipper, G1, or G3 by quantifying Jurkat T cell cleavage of the caspase-8 substrate, IETD-*p*-nitroanilide. At 24 h, cells treated with 5 μM G3 and 0.5 μM G1/G3 Zipper demonstrated comparable caspase-8 activity, whereas cells treated with 5 μM G1 demonstrated significantly weaker caspase-8 activity (Figure 3A).

We characterized caspase-3 activity induced by G1/G3 Zipper, G1, or G3 by quantifying Jurkat T cell cleavage of the substrate, Z-DEVD-AMC. At 4 h, cells treated with 0.5 μM G1/G3 Zipper, 5 μM G1, or 5 μM G3 demonstrated no measurable caspase-3 activity relative to untreated cells (Supplementary Figure 1). At 24 h, cells treated with 5 μM G3 demonstrated high caspase-3 activity. In contrast, cells treated with 0.5 μM G1/G3 Zipper demonstrated moderate caspase-3 activity, while cells treated with 5 μM G1 had low caspase-3 activity (Figure 3B).

### The Intracellular Mediators Involved in G1/G3 Zipper Pro-apoptotic Signaling Differ Relative to G1 and G3

We first evaluated the involvement of caspase-8 in Jurkat T cell death induced by G1/G3 Zipper, G1, or G3 using the caspase-8 inhibitor, Z-IETD-FMK. Inhibiting caspase-8 significantly reduced the activity of 5 μM G1 over 4 and 24 h (Figure 3C, Supplementary Figure 3). Inhibiting caspase-8 diminished the activity of 5 μM G3 over 24 h (Figure 3C), albeit to a lesser extent than G1, yet had only a weak effect on G3 over 4 h (Supplementary Figure 3). In contrast, inhibiting caspase-8 had no significant effect on 0.5 μM G1/G3 Zipper activity over 4 or 24 h (Figure 3C, Supplementary Figure 3).

Next, we evaluated the involvement of caspase-3 in Jurkat T cell death induced by G1/G3 Zipper, G1, or G3 using a commercially available caspase-3 inhibitor. Inhibiting caspase-3 significantly decreased the extent of cell death induced by 5 μM G1, 5 μM G3, or 0.5 μM G1/G3 Zipper over 24 h (Figure 3D). Notably, the caspase-3 inhibitor decreased the activity of G1/G3 Zipper, G1, and G3 to a similar extent. In contrast, inhibiting caspase-3 decreased the activity of G1/G3 Zipper over 4 h, yet had no effect on G1 or G3 (Supplementary Figure 3).

We characterized the role of ERK in Jurkat T cell death induced by G1/G3 Zipper, G1, or G3 using the ERK inhibitor, U0126. Inhibiting ERK increased the extent of cell death induced by G1/G3 Zipper, G1, or G3 over 4 h (Supplementary Figure 3). Over 24 h, inhibiting ERK also increased the pro-apoptotic activity of 0.5 μM G1/G3 Zipper (Figure 3E). In contrast, inhibiting ERK decreased the activity of 5 μM G1 over 24 h, but had no significant effect on the activity of 5 μM G3 (Figure 3E).

We characterized the role of JNK activation in Jurkat T cell death induced by G1/G3 Zipper, G1, or G3 using the ATP-competitive JNK inhibitor, SP600125 (“JNK inhibitor”). Inhibiting JNK activation significantly increased the extent of cell death induced by G3 and weakly increased the extent of cell death induced by G1/G3 Zipper over 4 h, whereas it had no effect on G1 activity over 4 h (Supplementary Figure 3). In contrast, inhibiting JNK activation decreased the extent of cell death induced by 5 μM G1, 5 μM G3, or 0.5 μM G1/G3 Zipper over 24 h (Figure 3F). Notably, inhibiting JNK decreased the pro-apoptotic activity of G3 over 24 h more than the activity of G1/G3 Zipper and G1, which were inhibited to a similar extent.

Collectively, these data suggest that the intracellular mediators that signal apoptosis downstream of G1/G3 Zipper, G1, and G3 differ relative to each other, as well as with time. In particular, caspase-8 was required to signal apoptosis via G1 over 4 h, while ERK activation was inhibitory. Caspase-3 and JNK activation played no role in G1 signaling at this time point. In contrast, over 24 h, caspase-8, caspase-3, ERK, and JNK all contributed to Jurkat T cell death via G1. Over 24 h, both JNK activation and ERK were inhibitory downstream of G3, whereas caspase-8 and caspase-3 were not involved. In contrast, caspase-8, caspase-3, and JNK contributed to pro-apoptotic signaling via G3 over 24 h, whereas ERK was not involved. Caspase-8 was never involved in pro-apoptotic signaling downstream of G1/G3 Zipper, whereas caspase-3 was involved at both early and late time points. Further, inhibiting ERK always amplified pro-apoptotic signaling downstream of G1/G3 Zipper, whereas JNK activation inhibited apoptosis induced by G1/G3 Zipper early but amplified it late.

### G1/G3 Zipper-Induced Cell Death Is Mediated by CD45 and Hindered by CD7

**Figure 4:**
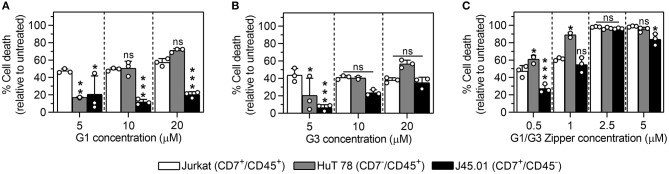
Comparison of Jurkat, HuT 78, and J45.01 T cell death via G1/G3 Zipper, G1, or G3. Percentage of dead cells following treatment of Jurkat, HuT 78, or J45.01 T cells with **(A)** 5–20 μM G1, **(B)** 5–20 μM G3, or **(C)** 0.5–5 μM G1/G3 Zipper for 24 h relative to untreated cells. For **(A–C)**, * indicates significant differences relative to Jurkat T cells; “ns” indicates no significant difference between cells. In all panels, one symbol denotes *p* < 0.05, two symbols denote *p* < 0.01, and three symbols denote *p* < 0.001. All data shown are *N* = 3, mean ± standard deviation, and tested for statistically significant differences using one-way ANOVA with Tukey's *post-hoc*.

We treated Jurkat (CD7^+^, CD45^+^), HuT 78 (CD7^−^, CD45^+^), and J45.01 (CD7^+^, CD45^−^) T cells with a range of G1/G3 Zipper, G1, or G3 concentrations to characterize the role of the cell surface glycoprotein receptors CD7 and CD45 in galectin-induced cell death. At 5 μM, G1 induced significantly less death of HuT 78 and J45.01 T cells than Jurkat T cells (Figure 4A). The extent of HuT 78 T cell death increased with G1 concentration (Figure 4A, gray bars), reaching a maximum that was slightly higher, although not significantly greater, than the extent of Jurkat T cell death induced by G1. In contrast, G1 only weakly induced J45.01 cell death at the concentrations tested (Figure 4A, black bars).

At 5 μM, G3 induced significantly less death of HuT 78 and J45.01 T cells than Jurkat T cells (Figure 4B), similar to G1. The extent of HuT 78 T cell death increased with G3 concentration (Figure 4B, gray bars), reaching a maximum that was slightly higher, although not significantly greater, than the extent of Jurkat T cell death induced by G3, similar to G1. In contrast to G1, the extent of J45.01 T cell death also increased with G3 concentration (Figure 4B, black bars), reaching a maximum that was comparable to the extent of Jurkat T cell death induced by G3.

At low concentrations, G1/G3 Zipper induced significantly more death of HuT 78 T cells than Jurkat T cells (Figure 4C, gray bars), similar to G1 and G3 at high concentration. At 0.5 μM, G1/G3 Zipper induced significantly less J45.01 T cell death than Jurkat T cell death, whereas the extent of J45.01 T cell and Jurkat T cell death at all other G1/G3 Zipper concentrations were similar.

Collectively, these data suggest that G1/G3 Zipper, G1, and G3 require CD45 to signal apoptosis. These data also suggest that CD7 antagonizes G1/G3 Zipper signaling at low galectin concentration, and may also inhibit G1 and G3 signaling at high galectin concentrations, whereas it is necessary for G1 and G3 signaling at low galectin concentrations.

## Discussion

Here we report a comparison of the pro-apoptotic signaling pathways activated by a synthetic G1/G3 tetramer referred to as G1/G3 Zipper, G1, and G3. Collectively, the data demonstrate that each protein differentially employs CD45, CD7, caspase-8, caspase-3, ERK, and JNK to induce apoptosis. In particular, G1/G3 Zipper, G1, and G3 all depend on caspase-3 to induce apoptosis. However, G3 induces significantly greater caspase-3 activity than G1/G3 Zipper or G1. Further, caspase-3 inhibition has a similar effect on G1/G3 Zipper and G1 activity, which is greater than the effect on G3 activity. Taken together, these observations suggest that G1/G3 Zipper behaves more like G1 than G3, in agreement with our initial hypothesis.

With regard to caspase-8, however, G1/G3 Zipper differs from both G1 and G3. For example, while G1/G3 Zipper, G1, and G3 all activate caspase-8, G1/G3 Zipper does not depend on it to induce apoptosis. In contrast, G1 depends on caspase-8 at both early and late time points, whereas G3 depends on it only at a later time point.

The role of JNK activation in G1/G3 Zipper signaling is more similar to its role in G3 signaling than G1 signaling. In particular, inhibition of JNK activation weakly increased the activity of G1/G3 Zipper at an early time point, but weakly decreased it at a late time point. In contrast, JNK inhibition strongly potentiated G3 activity at an early time point and significantly decreased it at a late time point, whereas JNK inhibition only decreased G1 activity at a late time point. This latter observation was consistent with a previous report which demonstrated that G1 induces Jurkat T cell death via activation of JNK and, in turn, c-Jun and AP-1 (Brandt et al., 2010). These observations suggest that G1/G3 Zipper does not induce apoptosis exclusively through G1-related pathways, which contrasts with our hypothesis. Instead, these data suggest that the G3 domains of G1/G3 Zipper are active as extracellular signals, but that their activity is diminished when compared to the native protein, which may be due to differences in carbohydrate-recognition domain valency.

Unexpectedly, ERK activation has contrasting roles in G1/G3 Zipper, G1, and G3 signaling. In particular, inhibiting ERK increased G1 activity early but diminished it at a later time point, increased G3 activity early but had no effect later, and increased G1/G3 Zipper activity at both early and late time points. These observations suggest that pro-apoptotic signaling via G1/G3 Zipper is not simply an additive combination of the signaling pathways activated by G1 and G3, but instead may involve an alternative pathway that integrates aspects of the pathways activated by both galectins.

Differences in the pro-apoptotic signaling pathways activated by G1/G3 Zipper, G1, and G3 may stem from differences in the role of the membrane glycoproteins CD7 and CD45 as signal transducers. For example, high CD45 expression was required for G1 activity under all conditions tested, whereas the activity of G1/G3 Zipper and G3 toward CD45 low cells increased with their concentration. Toward CD7 low cells, the activity of both G1 and G3 increased with their concentration, but was weakened relative to their activity toward CD7 high cells at low galectin concentration. In contrast, low CD7 expression enhanced G1/G3 Zipper activity at low concentrations, whereas it had no effect at high G1/G3 Zipper concentrations. Collectively, these data suggest that CD7 is not directly involved as a signal transducer of G1/G3 Zipper, but instead may act as an antagonist, whereas it is involved in both G1 and G3 signaling, as suggested in prior reports (Pace et al., 1999, 2000; Fukumori et al., 2003; Stillman et al., 2006). Note that any differences in the role of CD7 in galectin signaling observed here and reported elsewhere may be due to differences in protein concentration, culture time, or cell type which often vary across studies. Further, these data suggest that G1/G3 Zipper signals at least in part through CD45, which could be mediated by either the G1 or G3 domain, as both have been implicated in G1 and G3 signaling previously (Nguyen et al., 2001; Stillman et al., 2006). However, the observation that G1/G3 Zipper induces considerable death of CD45 low cells at all concentrations tested suggests that other membrane glycoproteins likely also act as signal transducers or binding partners. Future research efforts will work to identify membrane glycoproteins that are recognized by G1/G3 Zipper, as well as those that act as transducers of pro-apoptotic signals.

Both caspase-8 and JNK activation can induce changes in metabolic activity that lead to pro-apoptotic signaling (Li et al., 1998; Dhanasekaran and Reddy, 2017). We previously reported that G1/G3 Zipper decreased NADH content of Jurkat T cells to a greater extent than both native G1 and a polymer-stabilized G1 homodimer, as measured by conversion of resazurin to resorufin with the CellTiter-Blue^®^ cell viability assay (Promega) (Fettis et al., 2019). Here we observed that both caspase-8 and JNK activation are involved in pro-apoptotic signaling via G1. In contrast, pro-apoptotic signaling via G1/G3 Zipper depends on JNK activation but not caspase-8 activity. The observation that G1/G3 Zipper induced higher caspase-8 activity than G1 suggests that the increased metabolic dysfunction induced by G1/G3 Zipper relative to G1 reported previously is due to caspase-8. But why is caspase-8 a bystander in pro-apoptotic signaling downstream of G1/G3 Zipper but not G1? The answer may lie in the observed disparity in the role of ERK downstream of G1/G3 Zipper and G1. JNK and/or caspase-8 acting on mitochondria can lead to activation of caspase-9 that amplifies cell death by increasing caspase-3 activity (Li et al., 1998; Dhanasekaran and Reddy, 2008). Consistent with this, we observed higher caspase-3 activity in cells treated with G1/G3 Zipper relative to cells treated with G1. Further, phosphorylation via ERK inhibits caspase-9 (Allan et al., 2003), and here inhibiting ERK activity increased the extent of cell death induced by G1/G3 Zipper. Collectively, these observations suggest a role for caspase-9 downstream of mitochondrial dysfunction as a contributor to G1/G3 Zipper pro-apoptotic signaling. Future work will take a closer look at the interplay between JNK activation, mitochondrial function, ERK activity, and caspase−8,−9, and−3 in pro-apoptotic signaling induced by G1/G3 Zipper.

## Conclusions

G1/G3 Zipper, a synthetic engineered tetramer with two G1 and two G3 domains, induces apoptosis to a greater extent than G1 and G3 alone or in combination. The results reported here suggest that G1/G3 Zipper signals apoptosis through a pathway that depends on caspase-3 and JNK, like G1 and G3, but is independent of caspase-8 activity and is amplified by ERK inhibition, which contrasts with G1 and G3. G1/G3 Zipper signaling is mediated in part by CD45, similar to G1 and G3, but inhibited by CD7, which contrasts with G1 and G3. Collectively, these observations call into question our prior mechanistic hypothesis which suggested that G1 acts as the signal while G3 acts as an anchor. Instead, G1/G3 Zipper amplifies pro-apoptotic signaling by integrating the activities of both G1 and G3. Although we have not yet elucidated the particular intracellular signals through which G1/G3 Zipper induces apoptosis in exhaustive detail, we have begun to understand the roles of common pro-apoptotic signaling molecules in this pathway. Further, we have identified key differences between an engineered galectin variant and the native proteins from which it was created. Finally, we demonstrated that co-integrating galectins into a single construct can enhance their signaling activity. Increasing understanding of the interplay between G1 and G3 assembled into non-native architectures may provide new opportunities to therapeutically regulate innate and adaptive immunity.

## Data Availability Statement

The raw data supporting the conclusions of this article will be made available by the authors, without undue reservation, to any qualified researcher.

## Author Contributions

GH designed experiments, analyzed data, and wrote the paper. SF designed and conducted flow cytometry experiments, analyzed data, and contributed to writing and editing of the paper. MF designed and conducted caspase activity experiments, analyzed data, and edited the paper. RL assisted with collection and analysis of flow cytometry data and edited the paper.

### Conflict of Interest

The authors declare that the research was conducted in the absence of any commercial or financial relationships that could be construed as a potential conflict of interest.
