# Supplementary material for: A Synthetic Tetramer of Galectin-1 and Galectin-3 Amplifies Pro-apoptotic Signaling by Integrating the Activity of Both Galectins
Source: Front Chem. 2020 Jan 10;7:898. doi: 10.3389/fchem.2019.00898 (PMC6966408; doi:10.3389/fchem.2019.00898)
Supplement: Supplementary file 1 [file Data_Sheet_1.pdf]

Supplementary data

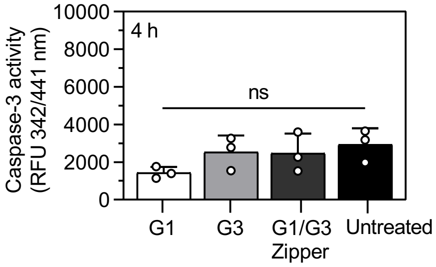

Supplementary Figure 1 – Quantification of caspase-3 activation in Jurkat T cell apoptosis induced by G1/G3 Zipper, G1, or G3 for 4 h. Quantification of caspase-3 activity in Jurkat T cells treated with 5 μM G1, 5 μM G3, or 0.5 μM G1/G3 Zipper for 4 h. “ns” indicates no significant difference relative to untreated. Data shown are *N* = 3, mean ± standard deviation, and tested for statistically significant differences using one-way ANOVA with Tukey’s *post-hoc*.

Supplementary data

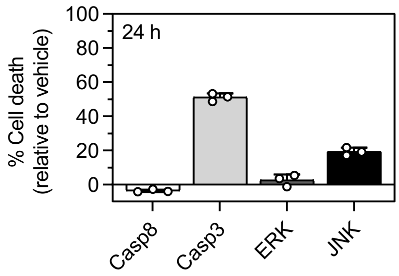

Supplementary Figure 2 – Quantification of Jurkat T cell death in the presence of inhibitor. Percentage of dead Jurkat T cells treated with caspase-8 (“casp8”) inhibitor (Z-IETD-FMK), caspase-3 (“casp3”) inhibitor I, ERK inhibitor (U0126), or JNK inhibitor II (SP600125) for 24 h, relative to 0.5% DMSO vehicle. Data shown are *N* = 3, mean ± standard deviation.

Supplementary data

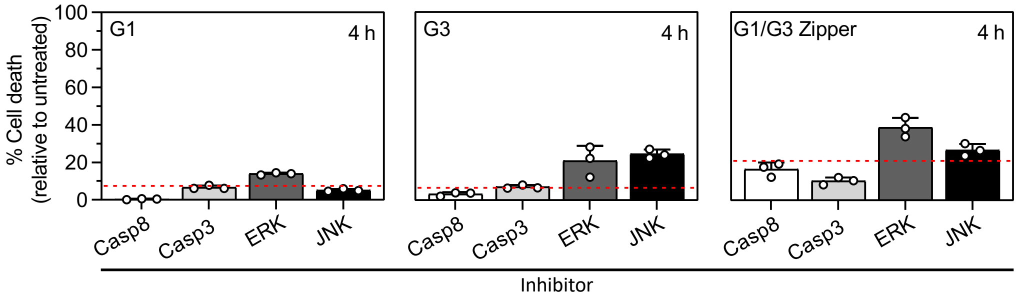

Supplementary Figure 3 – Characterization of the role of caspase-8, caspase-3, ERK, and JNK activation in Jurkat T cell apoptosis induced by G1/G3 Zipper, G1, or G3 at an early timepoint. Percentage of dead Jurkat T cells (relative to untreated cells) following treatment with caspase-8 ("casp8") inhibitor (Z-IETD-FMK), caspase-3 ("casp3") inhibitor I, ERK inhibitor (U0126), or JNK inhibitor II (SP600125) at 5 μM G1, 5 μM G3, or 0.5 μM G1/G3 Zipper for 4 h. For comparison, the red dashed lines in (C) indicates the percentage of dead Jurkat T cells treated with 5 μM G1, 5 μM G3, or 0.5 μM G1/G3 Zipper for 4 h, respectively, from Figure 2B. All data shown are *N* = 3, mean ± standard deviation.
